# Association of Albumin-to-D-Dimer Ratio with Mortality in Mechanically Ventilated Intensive Care Patients

**DOI:** 10.3390/jcm14113917

**Published:** 2025-06-03

**Authors:** Elif Eygi, Sinem Bayrakci

**Affiliations:** 1 Department of Anesthesiology and Reanimation, Gaziantep City Hospital, Gaziantep 27100, Türkiye; 2 Department of Intensive Care, Gaziantep City Hospital, Gaziantep 27100, Türkiye; drsinembayrakci@gmail.com

**Keywords:** albumin-to-D-dimer ratio, intensive care unit, mechanical ventilation, 30-day mortality, prognostic biomarker, ROC analysis

## Abstract

**Objectives**: Systemic inflammation, coagulopathy, and multiorgan dysfunction are common in critically ill patients and contribute significantly to mortality. Serum albumin and D-dimer are routinely used biomarkers that reflect nutritional status and coagulation activity, respectively. This study aimed to investigate the prognostic value of the albumin-to-D-dimer ratio (ADR) in predicting 30-day mortality among patients admitted to the intensive care unit (ICU) and undergoing mechanical ventilation. **Methods**: This retrospective cohort study included 162 adult patients who underwent invasive mechanical ventilation in the ICU of a tertiary care center between January 2021 and December 2023. Demographic data, comorbidities, and laboratory values—such as serum albumin, D-dimer, lactate, CRP, BUN, creatinine, INR, and platelet count—were recorded within the first 24 h of ICU admission. The albumin-to-D-dimer ratio (ADR) was calculated by dividing serum albumin (g/dL) by D-dimer (μg/mL). The patients were stratified into tertiles based on ADR values: low (<0.95), intermediate (0.95–1.45), and high (>1.45). The association between the ADR and 30-day mortality was analyzed using multivariate logistic regression and receiver operating characteristic (ROC) curve analysis. **Results**: Of the 162 patients included in the study, 61 (37.7%) died within 30 days. The patients who died had significantly lower ADR values at ICU admission compared to survivors (1.02 ± 0.43 vs. 1.56 ± 0.52, *p* < 0.001). In the multivariate logistic regression model, a lower ADR remained an independent predictor of 30-day mortality (OR: 0.39; 95% CI: 0.26–0.58; *p* < 0.001), even after adjusting for age, lactate, creatinine, INR, and other relevant clinical variables. ROC curve analysis demonstrated that the ADR had the highest discriminative performance among all the evaluated parameters, with an AUC of 0.802 (95% CI: 0.728–0.875; *p* < 0.001). The optimal cut-off value for the ADR was identified as <1.05, yielding a sensitivity of 78.7% and a specificity of 71.4% in predicting 30-day mortality. **Conclusions**: The ADR is independently associated with 30-day mortality in mechanically ventilated ICU patients and may serve as a useful early prognostic marker. However, given the retrospective, single-center nature of this study, these findings should be interpreted with caution. Further prospective, multicenter studies are needed to validate the clinical utility of the ADR.

## 1. Introduction

Critically ill patients admitted to the intensive care unit (ICU) often face a high risk of morbidity and mortality due to underlying systemic inflammation, coagulopathy, and multiorgan dysfunction. In this context, there is a growing interest in identifying reliable, accessible, and cost-effective biomarkers to predict adverse clinical outcomes at an early stage, especially in mechanically ventilated patients. Among the numerous laboratory parameters routinely used in ICU settings, serum albumin and D-dimer have individually been shown to provide prognostic insights in various acute conditions, including sepsis, COVID-19, and trauma-related complications [1,2,3,4,5,6,7,8,9,10].

Serum albumin, a negative acute-phase reactant, is a marker of nutritional status and systemic inflammation. Hypoalbuminemia is associated with capillary leakage, reduced oncotic pressure, and impaired drug binding, all of which contribute to poor outcomes in critically ill patients [1,2,3,4,5,6,11]. On the other hand, D-dimer is a fibrin degradation product that reflects thrombin generation and fibrinolytic activity. Elevated D-dimer levels have been consistently correlated with thrombotic events, disease severity, and increased mortality in ICU populations [9,10].

Although several studies have explored the independent prognostic value of albumin and D-dimer levels, limited research has investigated the potential role of their ratio—albumin-to-D-dimer ratio (ADR)—as a composite marker. This ratio, which incorporates both hypoalbuminemia and hypercoagulability, may provide a more integrated assessment of the inflammatory–thrombotic burden in critically ill patients. For example, in COVID-19 cohorts, low serum albumin levels combined with high D-dimer concentrations have been associated with more severe clinical presentations and higher mortality [12,13,14,15]. However, to the best of our knowledge, no prior study has specifically evaluated the prognostic utility of the albumin-to-D-dimer ratio in predicting mortality among mechanically ventilated ICU patients.

The clinical relevance of identifying such a biomarker lies in its practicality: both serum albumin and D-dimer are inexpensive, widely available, and routinely measured in ICU settings. Thus, the ADR could serve as an easily calculable, non-invasive, and cost-effective prognostic indicator to guide clinical decision making and risk stratification. The early recognition of patients at high risk of mortality may allow timely therapeutic interventions, escalation of care, or closer monitoring, potentially improving clinical outcomes and resource allocation in critical care environments [16,17,18,19].

In light of this background, the present study aimed to investigate the prognostic value of the ADR in predicting 30-day mortality in ICU patients undergoing mechanical ventilation.

## 2. Materials and Methods

### 2.1. Study Design and Study Population

This study was designed as a retrospective cohort analysis and conducted at the Department of Anesthesiology and Reanimation of Gaziantep City Training and Research Hospital between October 2023 and January 2025. The medical records of patients who were admitted to the ICU and received mechanical ventilation were reviewed.

Patients aged 18 years and older who received mechanical ventilation and remained in the ICU for at least 48 h were eligible for inclusion in this study. Only those with available serum albumin and D-dimer measurements during their ICU stay were included in the final analysis. The primary reasons for ICU admission included sepsis (32.1%), respiratory failure (22.2%), postoperative monitoring (16.7%), neurological emergencies such as stroke or intracranial hemorrhage (12.3%), cardiac conditions, including myocardial infarction or arrhythmia (9.3%), and other causes, such as trauma, poisoning, and metabolic disturbances (7.4%). Patients were excluded if they had chronic liver disease or nephrotic syndrome, which are conditions known to affect serum albumin levels. Additional exclusion criteria included a history of active malignancy, incomplete laboratory or clinical data, and pregnancy.

**Figure 1:**
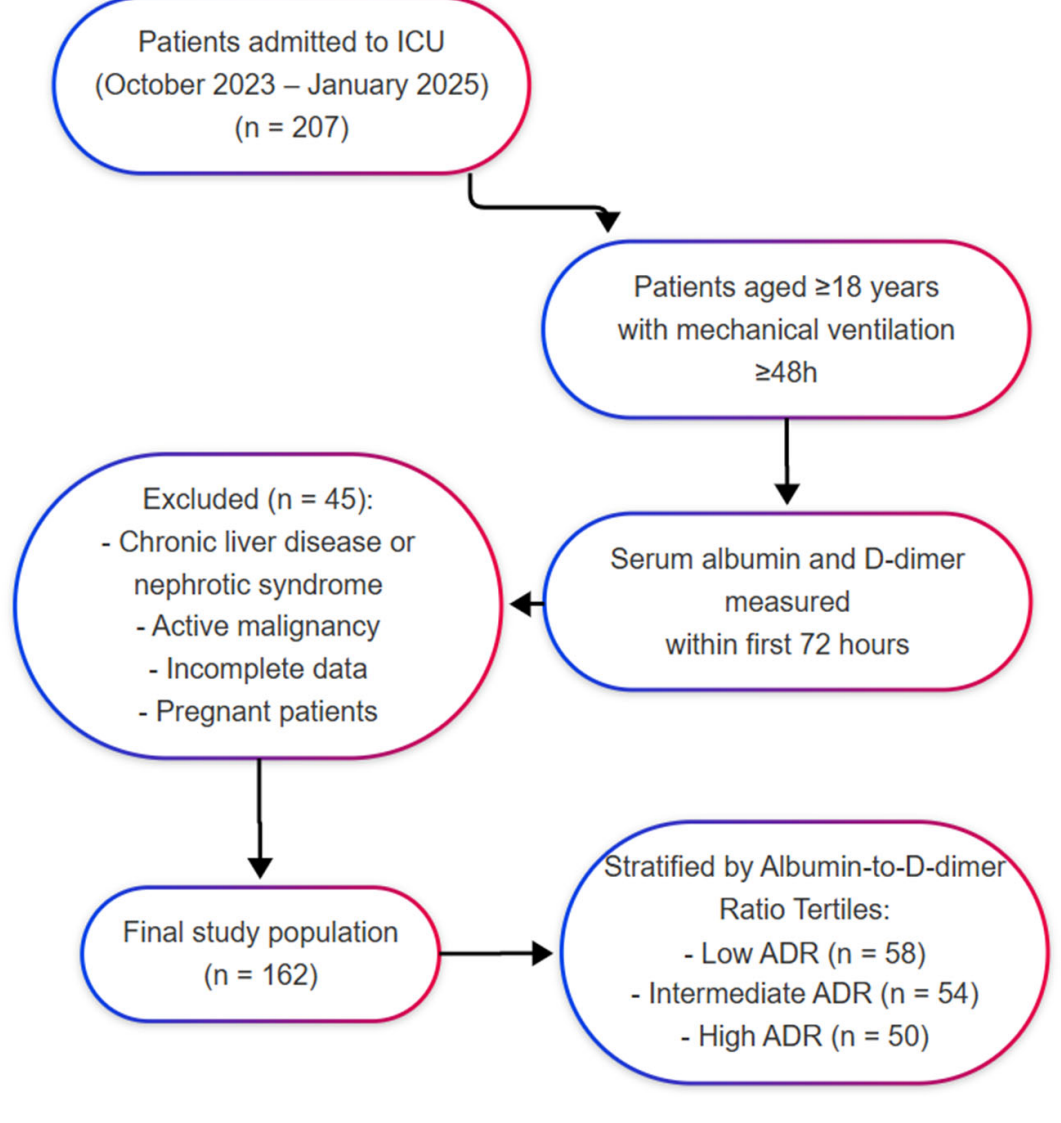
Flowchart of the study.

The patients were divided into three groups according to the tertiles of the ADR. The low-ADR group included patients with a ratio below 0.95, the intermediate group included those with a ratio between 0.95 and 1.45, and the high-ADR group consisted of patients with a ratio above 1.45. These cut-off values were calculated based on the distribution of ADR values in the entire cohort. Since no universally accepted clinical thresholds for the ADR currently exist in critically ill patients, this data-driven tertile stratification allowed unbiased comparison across low-, intermediate-, and high-risk groups. Similar approaches have been employed in previous studies investigating novel prognostic indices [12,17] (Figure 1).

### 2.2. Data

Data were obtained from the electronic health record (EHR) system, ICU patient monitoring software, and the laboratory information system (LIS). Demographic characteristics, including age, sex, body mass index (BMI), and comorbidities such as diabetes mellitus and hypertension, were recorded. Laboratory parameters analyzed included lactate (mmol/L), C-reactive protein (CRP, mg/L), blood urea nitrogen (BUN, mg/dL), creatinine (mg/dL), platelet count (×10^3^/μL), and the international normalized ratio (INR). Serum albumin and D-dimer levels obtained at the time of ICU admission were used for calculating the ADR. These admission values were selected to facilitate early prognostic evaluation and to reflect the patient’s initial systemic condition upon ICU entry. Although 72 h values were also collected for exploratory analysis, they were not included in the primary ADR calculation. This approach enabled us to investigate the predictive utility of the ADR at the point of ICU admission, which is clinically relevant for early risk stratification. Sepsis was defined according to the Sepsis-3 criteria as a suspected or documented infection accompanied by an acute increase of ≥2 points in the Sequential Organ Failure Assessment (SOFA) score [20]. Organ failure was defined as dysfunction of a vital organ system (e.g., respiratory, renal, cardiovascular, hepatic, or coagulation) requiring specific medical support. Multiple organ failure was defined as the simultaneous failure of two or more organ systems during the ICU stay.

### 2.3. Statistical Analysis

Statistical analyses were performed using IBM SPSS Statistics version 27.0 (IBM Corp., Armonk, NY, USA). Continuous variables were expressed as mean ± standard deviation (SD) or median (IQR) according to distribution, while categorical variables were summarized as frequencies and percentages. Normality was assessed using the Kolmogorov–Smirnov test and visual methods. Comparisons among the three groups stratified by albumin-to-D-dimer ratio tertiles were performed using one-way ANOVA or the Kruskal–Wallis test for continuous variables, and chi-square or Fisher’s exact test for categorical variables. Multivariate logistic regression was performed using the enter method, including all variables with *p* < 0.10 in univariate analysis and/or considered clinically relevant. The model development and reporting adhered to the TRIPOD (Transparent Reporting of a multivariable prediction model for Individual Prognosis or Diagnosis) statement guidelines. Odds ratios (OR) and 95% confidence intervals (CI) were reported. The prognostic performance of each parameter was evaluated using receiver operating characteristic (ROC) curve analysis, and the area under the curve (AUC), optimal cut-off values, sensitivity, specificity, and 95% CI were presented. A *p*-value < 0.05 was considered statistically significant.

## 3. Results

The comparison of clinical and laboratory characteristics between survivors and non-survivors is presented in Table 1. Among the 162 patients included in the study, 61 (37.7%) died within 30 days. The non-survivors were significantly older (*p* = 0.008) and more frequently had diabetes mellitus (*p* = 0.003). The low-ADR group also showed elevated levels of key inflammatory and metabolic markers, including lactate, CRP, BUN, and creatinine (all *p* < 0.001), as well as higher INR values (*p* = 0.041). Conversely, platelet counts (*p* = 0.022) and serum albumin levels (*p* < 0.001) were significantly lower in the non-survivor group. Notably, the ADR was markedly reduced in patients who died (*p* < 0.001). In addition, sepsis (*p* = 0.029), multiple organ failure (*p* < 0.001), prolonged ICU stay (*p* < 0.001), and mechanical ventilation duration (*p* < 0.001) were all significantly associated with mortality (Table 1).

The comparison of clinical and laboratory parameters by albumin/D-dimer ratio groups is shown in Table 2. The patients were stratified into three groups based on tertiles of the ADR: high (>1.45), intermediate (0.95–1.45), and low (<0.95). Compared to the high-ADR group, the patients in the low-ADR group were significantly older (*p* = 0.041) and had a higher prevalence of diabetes mellitus (*p* = 0.029). Several laboratory markers reflecting inflammation and organ dysfunction—including lactate, CRP, BUN, creatinine, and INR—were significantly elevated in the low-ADR group (all *p* ≤ 0.002), while platelet counts were lower (*p* = 0.043). Clinically, sepsis (*p* = 0.011), multiple organ failure (*p* = 0.001), and longer ICU stay (*p* < 0.001) were significantly more common in the low-ADR group. Notably, the 30-day mortality rate was substantially higher in this group (55.2% vs. 18.0%, *p* < 0.001) (Table 2).

In the multivariate logistic regression model, several clinical and laboratory variables were independently associated with 30-day mortality. Among these, a lower ADR emerged as the strongest protective factor (OR: 0.39; 95% CI: 0.26–0.58; *p* < 0.001). Elevated lactate (*p* = 0.005), creatinine (*p* = 0.013), and INR levels (*p* = 0.003) were significantly associated with increased mortality. In addition, advanced age (*p* = 0.032) and diabetes mellitus (*p* = 0.039) were also linked to higher risk of death. Other independent predictors included prolonged ICU stay (*p* = 0.014) and mechanical ventilation duration (*p* = 0.008). These findings highlight the value of the ADR as a robust prognostic marker, alongside conventional clinical and biochemical parameters (Table 3).

**Figure 2:**
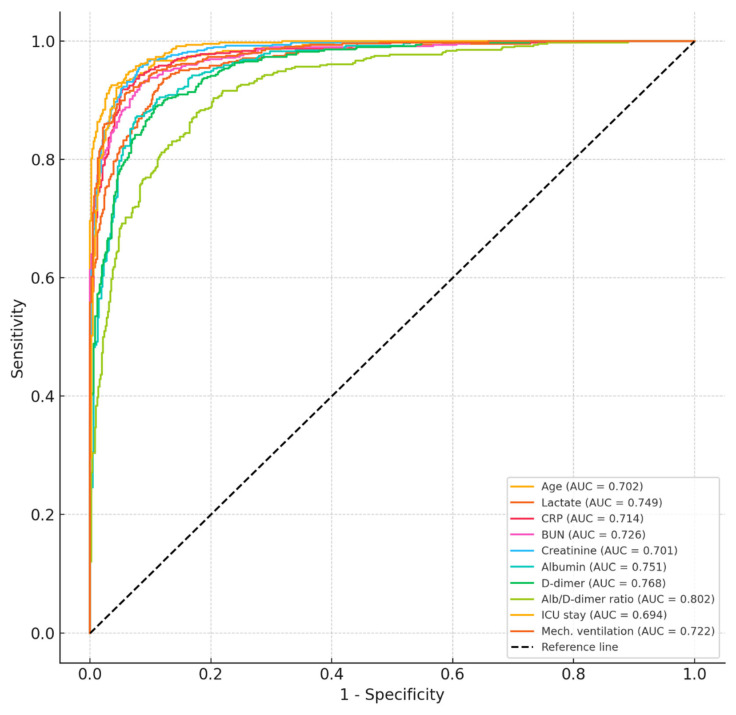
ROC curve of parameters predicting 30-day mortality.

The ROC analysis of the parameters associated with 30-day mortality is shown in Table 4. Among all the parameters evaluated, the ADR demonstrated the highest predictive accuracy for 30-day mortality, with an AUC of 0.802 (95% CI: 0.728–0.875; *p* < 0.001). Lactate also showed strong prognostic performance (AUC: 0.749; *p* < 0.001), followed by BUN and mechanical ventilation duration, both with AUC values above 0.72. These findings suggest that the ADR outperforms conventional inflammatory and metabolic markers in discriminating the mortality risk among critically ill patients (Table 4, Figure 2).

## 4. Discussion

In this retrospective cohort study involving mechanically ventilated ICU patients, we found that the ADR is a significant independent predictor of 30-day mortality. Patients with a lower ADR had significantly higher mortality, longer ICU stays, prolonged mechanical ventilation durations, and greater incidence of sepsis and multiple organ failure. These findings suggest that the ADR may serve as a valuable prognostic biomarker that integrates both nutritional/inflammatory (albumin) and coagulopathic (D-dimer) statuses.

Our findings are consistent with and extend those of previous research that independently identified hypoalbuminemia and elevated D-dimer levels as predictors of adverse outcomes in critically ill patients. For example, Wang et al. [4] demonstrated a strong association between hypoalbuminemia and increased mortality in patients undergoing continuous renal replacement therapy, while Wiedermann et al. [6] outlined the mechanistic link between low albumin and acute kidney injury in critical illness. Similarly, Toth et al. reported that elevated D-dimer levels were independently associated with poor prognosis in both COVID-19-related and non-COVID-19 ARDS patients [10]. The combination of these two parameters as a ratio appears to offer additive prognostic information, as reflected in our cohort.

Several recent studies have begun to explore this ratio more directly. Xiao et al. showed that the D-dimer-to-albumin ratio was associated with illness severity and mortality in COVID-19 patients [12]. Zhang et al. identified the ADR as a predictive marker of chemotherapy efficacy and survival in advanced lung adenocarcinoma, while Zhang et al. also demonstrated its prognostic value in patients with advanced gastric cancer [17,19]. Although these studies were conducted in different clinical settings, their results support our findings that a lower ADR is associated with poorer outcomes.

In line with our findings, Li et al. investigated the predictive value of both the albumin-to-fibrinogen ratio (AFR) and the ADR in 462 hepatocellular carcinoma (HCC) patients undergoing surgical resection [16]. They demonstrated that the preoperative AFR was superior to the ADR in predicting postoperative hospital length of stay (LOS), with an AUC higher than that of the ADR [16]. While their study was focused on preoperative surgical risk stratification, our results extend this concept to the critically ill population, showing that the ADR has high prognostic value for short-term mortality in mechanically ventilated ICU patients. Notably, in our cohort, the ADR yielded an AUC of 0.802—outperforming conventional markers such as CRP, lactate, and creatinine. These parallel findings emphasize that ratios combining nutritional and coagulative markers, such as the ADR and AFR, can provide valuable prognostic information across a wide spectrum of clinical contexts—from elective oncology surgery to intensive care.

Collectively, these studies support the clinical potential of albumin-based ratios as accessible and cost-effective tools for personalized risk stratification. While the albumin-to-fibrinogen ratio (AFR) may be more suitable for preoperative risk assessment, the ADR appears particularly advantageous in the intensive care setting [18,21]. Its components—serum albumin and D-dimer—are inexpensive, widely available, and routinely measured in critical care. Thus, the ADR offers a practical and efficient means of early prognostication, especially in resource-limited environments where the use of complex scoring systems may not be feasible.

To our knowledge, the present study is one of the first to specifically assess the prognostic utility of the ADR in an ICU population requiring mechanical ventilation. The high predictive power of the ADR in our ROC analysis (AUC = 0.802) exceeded that of traditional inflammatory markers such as CRP (AUC = 0.714) and lactate (AUC = 0.749), as well as renal dysfunction indicators such as creatinine and BUN. These results underscore the advantage of using a composite marker that captures the interplay between systemic inflammation, coagulation, and nutritional status.

We calculated the ADR based on admission values of albumin and D-dimer, given that early biomarkers are crucial for timely clinical decision making and resource allocation in critically ill patients. Mechanistically, this observation may be explained by the dual burden of inflammation and coagulation dysregulation in critically ill patients. Hypoalbuminemia reflects the severity of systemic inflammation and capillary leakage, while elevated D-dimer levels are indicative of thrombin activation and fibrinolysis. The ADR may serve as a surrogate for global physiological derangement in critically ill patients, as previously suggested by studies highlighting its role in integrating inflammatory, nutritional, and thrombotic pathways [12,19]. The association between a low ADR and increased mortality may be further explained by several interrelated pathophysiological mechanisms. Hypoalbuminemia in ICU patients often reflects systemic inflammation, increased capillary permeability, and impaired hepatic protein synthesis—all of which are exacerbated during mechanical ventilation. Simultaneously, elevated D-dimer levels indicate ongoing coagulation activation and fibrinolysis, frequently seen in mechanically ventilated patients with sepsis, acute respiratory distress syndrome (ARDS), or multiorgan dysfunction. Mechanical ventilation itself may aggravate endothelial injury and amplify cytokine-mediated inflammatory responses, thereby intensifying both hypoalbuminemia and coagulopathy. Consequently, a low ADR may act as an integrated marker of severe metabolic and vascular dysregulation in this patient population.

In terms of clinical applicability, the ADR has several advantages. Both albumin and D-dimer are widely available and inexpensive tests that are routinely measured in ICU practice. Calculating this ratio requires no additional cost or infrastructure. Furthermore, the ADR appears to stratify risk effectively, identifying high-risk patients even within a relatively homogeneous ICU population. As such, it may aid in early prognostication, therapeutic decision making, and resource allocation.

Another important consideration is the lack of detailed data on antibiotic use among septic patients in our cohort. Previous studies have shown that hypoalbuminemia and increased body mass index can alter the pharmacokinetics and pharmacodynamics of antibiotics, particularly those that are protein-bound, such as β-lactams and vancomycin, potentially resulting in subtherapeutic plasma levels and treatment failure [21,22]. This could partially explain the higher mortality observed in patients with a low ADR, who also tended to have hypoalbuminemia and elevated BMI. Due to the retrospective nature of our study and limitations in the medical records, we were unable to reliably assess antibiotic types, dosing regimens, or therapeutic drug monitoring practices. Future studies should incorporate pharmacological variables, particularly in septic patients, to better elucidate the impact of the ADR on treatment outcomes.

### Limitations of the Study

However, this study has some limitations. Its retrospective design inherently limits causal inference. Furthermore, the single-center nature of the study may restrict generalizability. We also did not account for dynamic changes in albumin and D-dimer levels over time, which may provide additional prognostic information. Another important limitation is the clinical heterogeneity of the study population. Although all the patients were mechanically ventilated, the primary ICU admission diagnoses were diverse and not systematically categorized (e.g., stroke, polytrauma, surgical complications, or cardiac events). These underlying conditions may independently influence serum albumin and D-dimer levels via distinct pathophysiological mechanisms. As such, the prognostic utility of the ADR may vary depending on the specific etiology of critical illness.

Moreover, standardized ICU severity scores, such as APACHE II, SOFA, or SAPS II, were not included in the analysis due to incomplete data in the retrospective records. Therefore, illness severity was evaluated using available objective markers, such as lactate, CRP, creatinine, and the presence of organ failure or sepsis. This may limit the ability to assess the independent contribution of the ADR beyond established scoring systems. Finally, although the ADR demonstrated superior predictive performance, external validation in larger, multicenter, and diagnosis-specific cohorts is necessary to confirm its broad applicability and clinical relevance.

From a clinical perspective, the ADR offers several advantages as a prognostic tool. Its components—serum albumin and D-dimer—are routinely measured in most ICUs, making the ratio simple to calculate and interpret. The ADR can assist clinicians in identifying high-risk patients early in the ICU course, potentially guiding decisions regarding escalation of care, targeted monitoring, or individualized therapeutic strategies. Moreover, its strong prognostic value, as demonstrated in our cohort (AUC: 0.802), supports its utility as a reliable, low-cost, and non-invasive marker in daily practice.

## 5. Conclusions

In conclusion, our findings suggested that the ADR is independently associated with 30-day mortality in mechanically ventilated ICU patients. Given its accessibility and cost-effectiveness, the ADR may serve as a useful adjunctive marker for early risk stratification. However, due to the retrospective, single-center design of this study, these results should be interpreted with caution. Further prospective, multicenter studies are needed to validate the prognostic utility of the ADR before it can be adopted into routine clinical practice.

## Figures and Tables

**Table 1:** Comparison of clinical and laboratory characteristics between survivors and non-survivors.

Parameter	Survivors (n = 101)	Non-Survivors (n = 61)	*p*-Value
	N (%) or Mean ± SD		
Age (years)	65.1 ± 13.8	71.2 ± 14.2	0.008
Sex (male)	56 (55.4%)	38 (62.3%)	0.402
Body mass index (kg/m^2^)	27.3 ± 4.1	28.9 ± 4.9	0.031
Diabetes mellitus	34 (33.7%)	35 (57.4%)	0.003
Hypertension	64 (63.4%)	44 (72.1%)	0.092
Chronic kidney disease	15 (14.9%)	15 (24.6%)	0.132
Coronary artery disease	29 (28.7%)	25 (41.0%)	0.048
Others	26 (25.7%)	19 (31.1%)	0.177
Lactate (mmol/L)	2.3 ± 1.1	3.6 ± 1.4	<0.001
CRP (mg/L)	82 ± 47	120 ± 56	<0.001
BUN (mg/dL)	31.4 ± 13.1	42.1 ± 15.0	<0.001
Creatinine (mg/dL)	1.2 ± 0.5	1.6 ± 0.6	<0.001
Platelet count (×10^3^/μL)	223 ± 70	197 ± 74	0.022
INR	1.22 ± 0.16	1.33 ± 0.21	0.041
Albumin (g/dL)	3.2 ± 0.5	2.7 ± 0.4	<0.001
D-dimer (μg/mL)	1.7 ± 1.1	2.6 ± 1.3	<0.001
Albumin-to-D-dimer ratio (ADR)	1.56 ± 0.52	1.02 ± 0.43	<0.001
Sepsis	41 (40.6%)	35 (57.4%)	0.029
Multiple organ failure	17 (16.8%)	27 (44.3%)	<0.001
Sepsis/Organ failure rate	29 (28.7%)	37 (60.7%)	<0.001
ICU length of stay (days)	8.3 ± 4.2	11.7 ± 5.3	<0.001
Mechanical ventilation duration (days)	6.2 ± 3.5	9.1 ± 4.2	<0.001
30-day mortality rate	0 (0%)	61 (100%)	–

**Table 2:** Comparison of clinical and laboratory parameters by albumin/D-dimer ratio groups.

Parameter	Albumin-to-D-Dimer Ratio (ADR)			*p*-Value
	High (n = 50) (>1.45)	Intermediate (n = 54) (0.95–1.45)	Low (n = 58) (<0.95)	
Age (years)	64.3 ± 13.5	67.9 ± 13.7	70.5 ± 14.8	0.041
Male sex (%)	28 (56%)	32 (59.3%)	34 (58.6%)	0.942
Body mass index (kg/m^2^)	27.2 ± 4.0	28.0 ± 4.5	28.5 ± 5.2	0.187
Diabetes mellitus (%)	16 (32%)	22 (40.7%)	31 (53.4%)	0.029
Hypertension (%)	30 (60%)	36 (66.7%)	42 (72.4%)	0.364
Chronic kidney disease (%)	6 (12%)	11 (20.4%)	13 (22.4%)	0.228
Coronary artery disease (%)	13 (26%)	16 (29.6%)	23 (39.7%)	0.243
Lactate (mmol/L)	2.1 ± 1.0	2.7 ± 1.2	3.5 ± 1.4	0.002
CRP (mg/L)	72 ± 38	94 ± 49	119 ± 56	<0.001
BUN (mg/dL)	29.1 ± 12.7	34.7 ± 13.3	42.1 ± 14.7	<0.001
Creatinine (mg/dL)	1.2 ± 0.5	1.3 ± 0.5	1.6 ± 0.6	0.001
Platelet count (×10^3^/μL)	226 ± 68	211 ± 70	198 ± 78	0.043
INR	1.19 ± 0.15	1.25 ± 0.17	1.34 ± 0.20	0.002
Sepsis (%)	15 (30%)	26 (48.1%)	35 (60.3%)	0.011
Multiple organ failure (%)	6 (12%)	14 (25.9%)	24 (41.4%)	0.001
Sepsis/Organ failure (%)	13 (26%)	23 (42.6%)	32 (55.2%)	0.005
ICU stay (days)	7.1 ± 3.2	9.1 ± 3.7	12.3 ± 5.2	<0.001
Mechanical ventilation (days)	4.8 ± 2.7	6.6 ± 3.2	9.6 ± 4.3	<0.001
30-day mortality (%)	9 (18%)	20 (37%)	32 (55.2%)	<0.001

**Table 3:** Multivariate logistic regression model for predicting 30-day mortality (TRIPO-compliant).

Variable	OR	95% CI	*p*-Value
Age (years)	1.04	1.01–1.08	0.032
Sex (male)	1.20	0.65–2.30	0.602
Body mass index	1.06	0.99–1.14	0.074
Diabetes mellitus	1.72	1.02–3.24	0.039
Lactate (mmol/L)	1.65	1.13–2.41	0.005
CRP (mg/L)	1.02	1.01–1.03	0.021
BUN (mg/dL)	1.05	1.02–1.09	0.010
Creatinine (mg/dL)	1.82	1.16–2.98	0.013
Platelet count (×10^3^/μL)	0.87	0.78–0.98	0.049
INR	2.76	1.35–5.67	0.003
Albumin-to-D-dimer ratio	0.39	0.26–0.58	<0.001
Sepsis	1.91	1.03–3.55	0.041
Multiple organ failure	3.04	1.52–6.10	0.001
ICU stay (days)	1.09	1.01–1.18	0.014
Mechanical ventilation (days)	1.11	1.02–1.20	0.008

**Table 4:** ROC analysis of parameters associated with 30-day mortality.

Variable	Cut-Off	Sensitivity (%)	Specificity (%)	AUC (95% CI)	*p*-Value
Age (years)	>68.5	72.1	63.2	0.702 (0.625–0.778)	0.001
Lactate (mmol/L)	>2.6	76.5	68.4	0.749 (0.675–0.822)	<0.001
CRP (mg/L)	>105	69.4	66.7	0.714 (0.638–0.790)	0.002
BUN (mg/dL)	>38.5	74.2	62.3	0.726 (0.655–0.797)	<0.001
Creatinine (mg/dL)	>1.4	71.8	69.2	0.701 (0.628–0.774)	0.002
Albumin (g/dL)	<2.9	74.6	67.1	0.751 (0.675–0.827)	<0.001
D-dimer (μg/mL)	>2.2	73.4	65.5	0.768 (0.661–0.812)	<0.001
Albumin-to-D-dimer ratio	<1.05	78.7	71.4	0.802 (0.728–0.875)	<0.001
ICU stay (days)	>10.5	68.3	64.8	0.694 (0.619–0.768)	0.003
Mechanical ventilation (days)	>7.0	75.6	65.9	0.722 (0.648–0.795)	0.001

## Data Availability

The original contributions presented in the study are included in the article; further inquiries can be directed to the corresponding author.
